# Interaction between bacterial endophytes and host plants

**DOI:** 10.3389/fpls.2022.1092105

**Published:** 2023-01-18

**Authors:** Sehrish Mushtaq, Muhammad Shafiq, Muhammad Rizwan Tariq, Adnan Sami, Muhammad Shah Nawaz-ul-Rehman, Muhammad Hamza Tariq Bhatti, Muhammad Saleem Haider, Saleha Sadiq, Muhammad Taqqi Abbas, Mujahid Hussain, Muhammad Adnan Shahid

**Affiliations:** ^1^ Faculty of Agricultural Sciences, University of the Punjab, Lahore, Pakistan; ^2^ Department of Horticulture, Faculty of Agricultural Sciences, University of the Punjab, Lahore, Pakistan; ^3^ Department of Food Science, Faculty of Agricultural Sciences, University of the Punjab, Lahore, Pakistan; ^4^ Department of Plant Breeding and Genetics, Faculty of Agricultural Sciences, University of the Punjab, Lahore, Pakistan; ^5^ Virology Lab, Centre of Agricultural Biochemistry and Biotechnology (CABB), University of Agriculture Faisalabad Pakistan, Faisalabad, Pakistan; ^6^ Institute of Biochemistry, Biotechnology, and Bioinformatics (IBBB), The Islamia University of Bahawalpur, Bahawalpur, Pakistan; ^7^ Department of Plant Pathology, Faculty of Agricultural Sciences, University of the Punjab, Lahore, Pakistan; ^8^ Horticultural Science Department, North Florida Research and Education Center, University of Florida/IFAS, Quincy, FL, United States

**Keywords:** host endosymbiont interactions, mechanism of interaction, bacterial endophytes, plants, endophytic

## Abstract

Endophytic bacteria are mainly present in the plant’s root systems. Endophytic bacteria improve plant health and are sometimes necessary to fight against adverse conditions. There is an increasing trend for the use of bacterial endophytes as bio-fertilizers. However, new challenges are also arising regarding the management of these newly discovered bacterial endophytes. Plant growth-promoting bacterial endophytes exist in a wide host range as part of their microbiome, and are proven to exhibit positive effects on plant growth. Endophytic bacterial communities within plant hosts are dynamic and affected by abiotic/biotic factors such as soil conditions, geographical distribution, climate, plant species, and plant-microbe interaction at a large scale. Therefore, there is a need to evaluate the mechanism of bacterial endophytes’ interaction with plants under field conditions before their application. Bacterial endophytes have both beneficial and harmful impacts on plants but the exact mechanism of interaction is poorly understood. A basic approach to exploit the potential genetic elements involved in an endophytic lifestyle is to compare the genomes of rhizospheric plant growth-promoting bacteria with endophytic bacteria. In this mini-review, we will be focused to characterize the genetic diversity and dynamics of endophyte interaction in different host plants.

## 1 Introduction

Plants interact with diverse microbial populations in the ecosystem (Delaux et al., 2015). Microorganisms can colonize on plants’ surfaces or internal parts depending on the host genotype and the molecular signals released by plant roots. Microorganisms can colonize on plants’ surfaces or internal parts depending on the host genotype and the molecular signals released by plant roots. Endophytes are prokaryotic bacteria found within the healthy host tissue (Brader et al., 2014). Bacterial endophytes can benefit the host in several ways, such as biotic and abiotic stress resistance, increased availability of nutrients, degradation of toxic molecules, and production of phytohormones (Kandel et al., 2015).

Plant population dynamics have soil microbial intermediation. The plant has a microbial population in the phyllosphere, endophytes, or rhizospheric microbes. The ecology and phenotype of the plants can be affected by the influence of symbiotic microbes on the atmosphere and competition for soil resources.

The plant genotype affects the microbial make-up of the phyllosphere, rhizosphere, and endophytic microorganisms (Lynch et al., 2001). Although the precise method involves the plant-associated microorganisms and ecosystem function, the other specific mechanism is still unknown. Because they are co-evolved with bacteria, plants are immobile and need to control the results of their intricate interactions (Schnitzer and Klironomos, 2011). Different sorts of chemicals are continuously produced by plant roots, gathered, and secreted into the soil (Wood et al., 2012) known as the root exudates which contain enzymes, water, mucilage, H^+^ ions, and primary, secondary compounds made up of carbon (Singh, 2015). Every plant species’ rhizosphere is known to have a microorganism population that is 100 times higher than soil and is mostly controlled by compounds generated by roots (Jonkers et al., 2003; Bever, 2003). The favorable plant-soil microbial response enhances the microbial populations’ spatial spread (Schimel et al., 2007), while negative reaction results in plant replacement, which demands recolonization of locally specific roots (Bever et al., 2010; Pedrotti et al., 2013).

It has been proposed that endophytic bacteria vary from rhizobacteria in their genetic architecture, which may account for their capacity to colonise plant tissues internally. However, no specific gene or gene family has been found to explain the endophytic regime. In a 2014 study, the whole genomes of nine Proteobacteria were compared to identify a list of genes that may play a role in the endophytic activity. So yet, only a few of those genes have undergone experimental testing to determine whether they are involved in endophytic colonisation (Shen et al., 2013; Ouyabe et al., 2019). In this study, we have documented some mechanisms involved in plant endophyte interaction at the molecular level.

## 2 Plant growth promotion by endophytes

PGPEs enhance plant development through three interconnected mechanisms: phytostimulation, biofertilization, and biocontrol. Phytostimulation is the production of phytohormones for direct plant development (Vishwakarma et al., 2021). The amount of the plant hormone ethylene frequently declines as a result of the enzyme 1-aminocyclopropane-1-carboxylate (ACC) deaminase (Cruz Barrera et al., 2020). According to numerous studies, the pea plant and the pepper plant (*Pseudomonas putida* and *Piper nigrum*, respectively) both have bacterial endophytes that release ACC deaminase to aid plant growth (Ruduś et al, 2013). By controlling ethylene levels in plants, ACC deaminize production may minimize abiotic stress because an increase in ethylene can obstruct DNA synthesis, root and shoot growth, and cell division. However, the specific method for enhanced plant development is still unknown (González Candia, 2021). Bacterial strains also produced other hormones which include abscisic acid, indole-3-acetic acid, and jasmonic acid, to stimulate plant growth (Forchetti et al., 2007).The endophytes can enhance plant growth by increasing the availability of important nutrients known as bio-fertilization.

Nitrogen fixation is the most studied phenomenon of bio-fertilization which is the conversion of atmospheric nitrogen into ammonia (Mishra and Arora, 2016). Bacterial species like *Azospirillum* spp., *Pantoea agglomerans*, and *Azoarcus* spp. all are known to be involved in a substantial amount of nitrogen fixation in plant roots (Indiragandhi et al., 2008). Nonetheless, only 21 PGPEs can increase plant phosphorus availability by solubilizing phosphate. The metal cation linked to phosphorous is chelated as a result of the release of low molecular weight acids, making it more available to plants. The researchers have isolated, identified, and assessed the ability of *Achromobacter xiloxidans* and *Bacillus pumilus* to solubilize phosphate in sunflowers (Barrera et al., 2020). PGPEs were utilized to treat corn, lowering the quantity of artificial phosphorus fertilizer required while increasing yields by up to 50% (Cruz Barrera et al., 2019).

The protection of plants from phytopathogens and their growth promotion is known as biological control. Antibiotic and siderophores production are involved in biological control mechanisms. Siderophores like pyochelin and alicyclic acid and chelate iron are not directly involved in disease control due to their competition with pathogens for trace metals (Leopold, 1964). The disease can be suppressed in plants by antimicrobial metabolites secreted by bacterial endophytes such as 2,4-diacetylphloroglucinol (DAPG). Seed treatment of eggplant *(Solanum melongena*) with DAPG-producing bacterial endophytes reduced 70% of eggplant wilt caused by *Ralstonia solanacearum* (Rana et al., 2020a).

*Burkholderia, Bacillus, Pseudomonas, Enterobacter*, and *Serratia* are just a few of the bacterial endophyte strains that are successful at preventing the growth of pathogenic germs in both *in vitro* and *in vivo* settings (Khan and Doty, 2009). Aside from that, bacteria from the genera *Bacillus, Enterobacter, Arthrobacter, Azotobacter, Isolptericola, Streptomyces*, and *Pseudomonas* improved the crop’s stress resistance from heat, drought, and salt (Rana et al., 2020b; Khalil et al., 2021). The most important interaction between these endophytes and symbiotic plants allowed the plants to significantly increase their biomass and height while lowering stress. Although, it is not yet clear how bacterial endophytes lessen abiotic stress (Liu et al., 2014).

### 2.1 Rhizobium and process of nodule formation

Rhizobium is a member of the family *Rhizobiaceae* and the class *Alphaproteobacteria*. Rhizobium, was the name given to this genus for the first time by Frank in 1889. There are 11 non-rhizobial species and 49 rhizobial species in the family *Rhizobiaceae* at the moment (Ledermann et al., 2021). The rhizobial species induce the nodules on the roots of plants (*Fabaceae* family) and are linked to symbiotic nitrogen-fixing bacteria. The nodule’s nitrogen fixation activity is extremely oxygen sensitive. The host plant receives continual supplies of reduced nitrogen from the bacterial enzyme system in this symbiotic connection, and the bacteria in exchange receive nutrients and energy from the plant (Van Rhijn and Vanderleyden, 1995). Nodules can occur in about 10% of legumes. The majority of the rhizobacteria in soil are oxygen sensitive and feed on the decomposing remains of other organisms.

**Figure 1:**
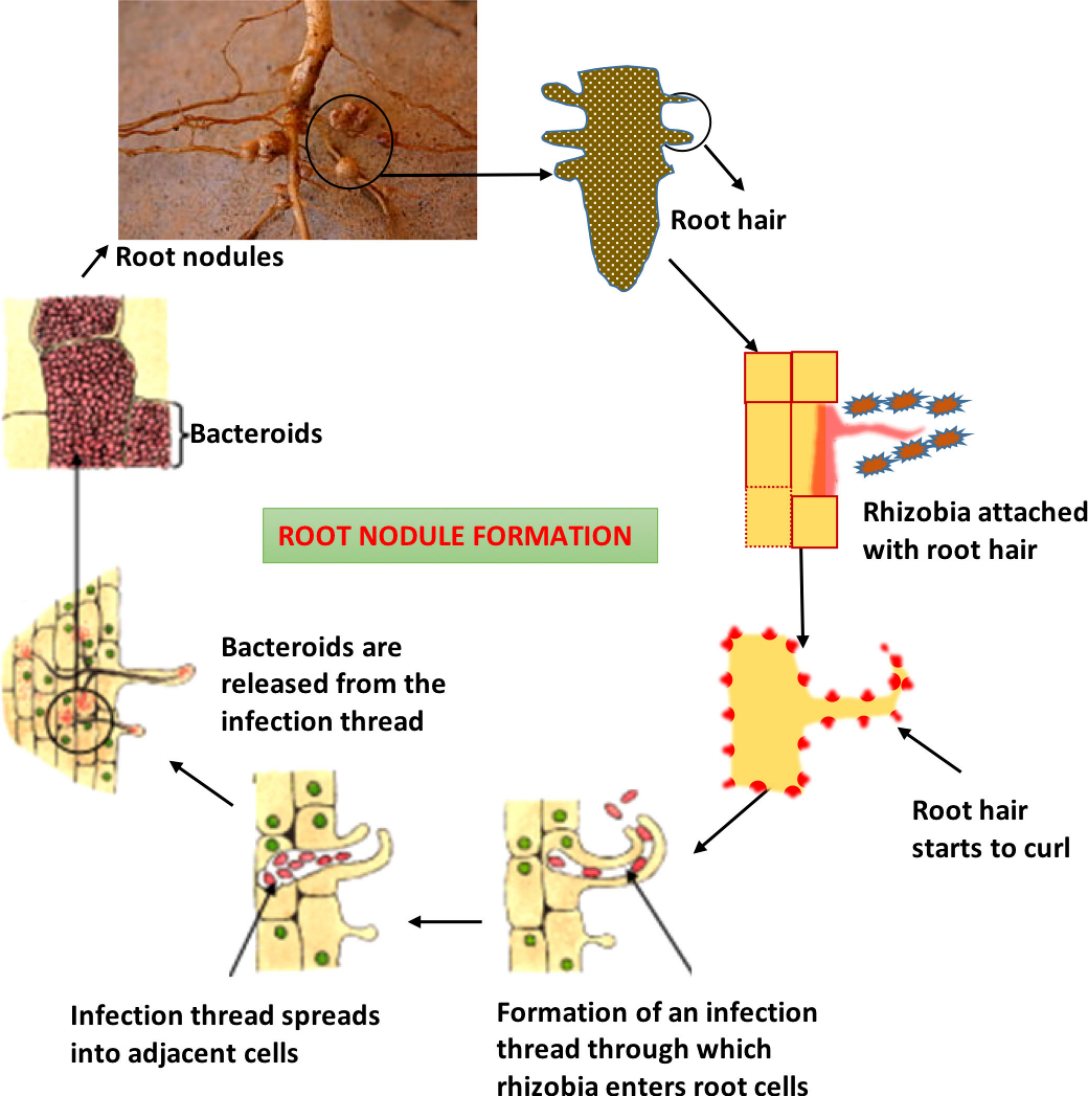
Diagrammatic representation of the whole process of nodule formation through rhizobia.

In roots, nitrogen-fixing bacteria occur as irregular cells known as bacteroids, which are frequently Y, club-shaped and appear as straight rods with a regular structure **(** **Figure 1** **)**. Bacteroidsencode genes that determine the rhizobium’s host specificity (Lodwig and Poole, 2003). Rhizobia that generate nodules but are unable to fix nitrogen are sometimes referred to as ineffective strains, whereas effective strains cause nitrogen fixation in nodules. Nodule development is controlled by certain genes known as nod genes i.e. nodF, nodE, nodL, nodP, nodQ, and nodH (Basile and Lepek, 2021). Some substances, such as flavonoids, are released by the root cells and trigger the production of nodules in bacteria by activating the nod gene. In essence, these chemicals are in charge of identifying the proper host and attaching to the root hairs.

The nod factors, which are secreted by bacteria, cause the root hairs to curl (Moran, 1997). The root hair tip is damaged by rhizobia, which also causes the infection thread to arise. The thread then extends to neighboring cells by thread branching, and the bacteria continue to grow within the growing network of tubes, continuing to create nod factors that encourage the growth of the root cells and ultimately result in the formation of root nodules (Oldroyd et al., 2011). Following a week of infection, nodules are visible with the unaided eye and each nodule contains thousands of living rhizobium bacteria, the majority of which are malformed and are referred to as bacteroids. Small sections of the plant cell membranes called symbiosomes, which may or may not include multiple bacteroids, are located next to bacteroids and are active sites for nitrogen fixation (Ratu et al., 2021). Through the *Nitrogenase enzyme*, also known as Nitrogenize catalysis, nitrogen gas from the atmosphere is converted inside legume nodules into ammonia, which is then assimilated into amino acids, DNA, and RNA as well as significant energy molecules like ATP or other chemicals like vitamins, flavones, and hormones (Bergersen, 1961). The Nitrogenize complex is protected by a variety of mechanisms used by aerobic free-living bacteria, including physical barriers and fast metabolic rates. Azotobacter, for instance, circumvents this issue by maintaining the lowest oxygen concentration in its cells and the greatest rate of respiration of any organism. In the instance of Rhizobium, the nodule’s red iron-containing protein, similar to hemoglobin in function to bond with oxygen, maintains control over the oxygen level (Lindström and Mousavi, 2020). However, this avoids the accumulation of free oxygen to prevent the loss of Nitrogenize activity while still providing enough oxygen for the metabolic functioning of bacteriods. Rhizobia and plants work together to make leghemoglobin, something neither of them could ever do on their own. Even in poor soil with few nutrients and insufficient nitrogen to support the growth of other plants, these nodules increase crop output (Lodwig and Poole, 2003).

### 2.2 Spread and variation of microbes from seed to plants

**Figure 2:**
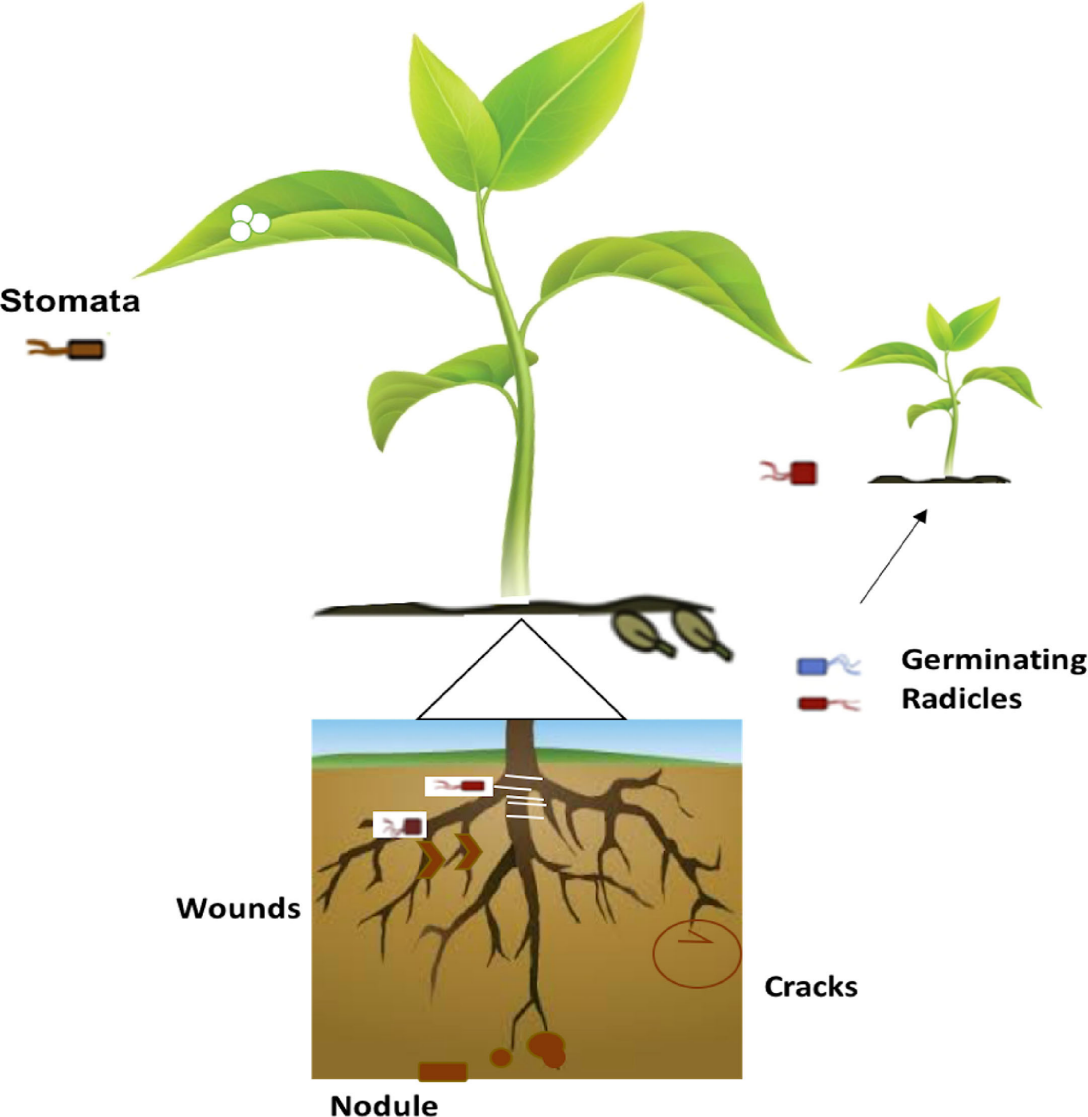
Overview of the endophytic bacterial mode of entry into different plant tissues.

Plants and their microbial diversity vary throughout their life span of plants. These factors, prompt the structure and variety of the microbial community (Honma and Shimomura, 1978). Seed-born microbes gain entry into the germinating plant and take advantage of other colonizing microbes as well as opportunistic pathogens from the surrounding soil (Glick et al., 1999; Oteino et al., 2015). Hence the overall microbial biota and population changed dramatically throughout the life cycle of plants. The important ways of entry into host plants are through root hair cells, root cracks, and wounds whereas other sources include stomata particularly of young stems and leaves; lenticels, and germinating radicles (**Figure 2**). Vertical seed transmission is another possible way to receive endophytic bacteria through plant host generations (Bergersen, 1961).

### 2.3 Presence of plant microbes in different parts of plants

Microorganisms associated with plants formed a complex network. Different studies suggested that plant-associated microbes live inside plant tissues or on the surface of plant parts such as leaves, stems, fruit, and roots (Clarholm, 1985). The microbiome studies of *A. thaliana* leaves showed that plant genotype, surrounding plants, and abiotic features affected the microbial population structure (Teixeira et al., 2013). These interactions are responsible for expediting the defense signals between plants and the efficacy of natural biological control agents (Morgan et al., 2005). Microbial populations might indirectly affect the other taxa of microbes by altering the host growth response or metabolites without direct interaction with microbes.

## 3 Beneficial effects of microbes on plant growth and development

Plants usually take nutrients from the soil which constitutes a pool for microscopic life forms including bacteria, fungi, actinomycetes, algae, and protozoa. So, among them, the bacteria are the most common ones and have the maximum proportion in soil. The maximum number of bacteria present in the rhizosphere near the roots of plants is different from bulk soil (Luu et al., 2020). As these bacteria are present in more concentration in the soil so the bacteria may affect a plant through three different pathways (Edwards and Harding, 2004). PGPEs can promote plant growth directly by expediting the procurement of compounds or modifying levels of plant hormones and reducing the inhibitory effect of plant growth and pathogenicity by acting as biocontrol agents (Yan et al., 2019). The benefits provided by the endophytes to the host plants and their mechanisms are described in (**Table 1**).

**Table 1:** Examples of plant growth-promoting rhizobacteria tested for various crop types.

PGPR	Plant	Benefits to plant growth	References
*Pseudomonas* sp.	Green gram	Increased plant dry weight, number of nodules, total chlorophyll content, root/shoot N, P seed protein, and yield.	(Del Carmen Orozco-Mosqueda et al, 2020)
	Soybean Wheat	Increased soil enzyme activity, nutrient absorption, and yield	(Kalyani et al., 2008)
	Chickpea	An enhanced fresh and dry weight of plants	(Berendsen et al., 2012)
	Rice	More ability to control fungal and bacterial pathogens	(Bulgarelli et al., 2012)
	Canola	Encouraged growth and cadmium accumulation in plants	(Agler et al., 2016)
	Mustard	Improved growth and reduced Cr contents among plants	(Foster, 1988)
	Soybean, mung bean, wheat	Promotes growth of plants	(Bertin et al., 2003)
*Pseudomonas putida*	Mung bean	The ethylene production repressed in treated plant Increase the growth and decreases Pb and Cd uptake	(Glick, 2012) (Ahemad and Khan, 2012)
	Lectuca	Enhancement of shoot/root length attained through concentrated inoculants	(Sharma et al., 2011)
	Artichoke	PSB along with N fixers increase in shoot length/weight, germination percentage seedling vigor, and reduction in germination time	(Tank and Saraf, 2010)
*Pseudomonas aeruginosa*	Maize	Endorsed plant growth and helped soil metal utilization, increase Pb and Cr uptake	(Lawongsa et al., 2008)
	Black gram	Reduced Cd deposition in tissues, widespread rooting, and increased plant growth	(Wu et al., 2015)
	Indian mustard and pumpkin	Increased in plant growth, decrease in Cd uptake	(Rajkumar et al., 2006)
	Tomato, Okra, African spinach	Increase in Dry weight of tomato, okra, and spinach	(Gupta et al., 2002)
*Pseudomonas fluorescens*	Alfalfa	Enhanced Fe and Cu movement from root/shoot	(Mayak et al., 1999)
	Peanut	Increase in pod yield and nodule dry weight	(Lobo et al., 2019)
	Soybean	Increased plant growth	(Rekha et al., 2007)
	Canola	Protect plants against the inhibitory effects of Cd	(Jahanian et al., 2012)
	Maize	Increase of plant growth, height, seed weight, no. of seed/ear, leaf area, shoot dry weight	(Curá et al., 2017)
*Azospirillum amazonense*	Rice	Grain dry matter deposition, panicle count, and nitrogen buildup at the grain maturity stage all increase	(Sant'anna et al, 2011)
*Azospirillum brasilense*	Common bean	Increase of Root growth in plants	(Adesemoye et al, 2008)
*Azospirillum lipoferum*	Cotton	An increase in soil microorganisms, plant height, and seed production was observed, but no changes in boll weight or staple length.	(Fayez and Daw, 1987)
*Azotobacter chroococcum*	Chinese mustard	Increased plant development and metal toxicity protection for the plant	(Jha, 2017)
*Azospirillum brasilense*	Rice	Increased grain yield	(Gupta et al., 2005)
*Kluyvera ascorbate*	Mustard, Tomato Canola,	Heavy metals reduce plant growth but do not boost metal uptake.	(Safronova et al., 2006)
*Bradyrhizobium*	Green gram	The development traits at all of the studied pesticide dosages (quizalafop-p-ethyl and clodinafop)	(Wani et al., 2007)
	Soybean and yellow Lupin	Increased biomass, nitrogen content, deposition of metals	(Dell’amico et al, 2008)
	Green gram	Increase of nodule number, seed yield, grain protein, root/shoot N at 290 mg Ni/kg soil	(Burd et al., 2000)
*Brevundimonas*	Canola	Isolated cadmium directly from the solution	(Gholami et al., 2009)
*Enterobacter cloacae*	Canola	Significant increases in root and shoot length were observed.	(Bashan and González, 1999)
*Klebsiella oxytoca*	Maize	Increase of plant growth parameters	(Remans et al., 2008)
*Enterobacter sakazakii*			
*Brevibacillus*	White clover	Increased plant growth and nutrition and decreased zinc conc.	Anjum et al., 2007)
*Methylobacterium oryzae, Berkholderia* sp.	Tomato	Significant increase in shoot/root length attained through bacterial cells inoculation	(Wu et al., 2006)
*Sinorhizobium* sp.	Brown mustard	Increased the efficacy of Pb	(Thakuria et al., 2004)
*Bacillus* spp	Barley	Increased root/shoot weight	(Dary et al., 2010)
*Rhizobium* sp.	Pea	Increase of the dry matter, nodule numbers, root/shoot nitrogen	(Lugtenberg and Kamilova, 2009)
*Mycobacterium* sp.	Canola	Prevent plant against the inhibitory effects of cadmium	(Wani et al., 2008)
*Bacillus* sp. *Paenibacillus* sp.	Rice	Considerably encouraged the root/shoot growth.	(Robinson et al., 2001)

## 4 Role of PGPEs against biotic stress

Throughout their lives, plants are exposed to harmful abiotic and biotic stresses. The damage that bacteria, fungi, viruses, nematodes, viroids, and insects do to plants is referred to as “biotic stress.” Rhizobacteria that promote plant growth by generating phytohormones or facilitating the uptake of particular nutrients might affect plant growth through biotic stress (Tiwari et al., 2020). However, PGPR reduces or even eliminate the negative impacts of plant pathogens. For example, *Pseudomonas fluorescens* produces 2,4-Diacetyl Phloroglucinol, which inhibits the development of pathogenic fungi in plants (Suslow and Schroth, 1982). Chitinase and laminarinase, two extracellular enzymes generated by *P. stutzeri*, caused the lysis of *Fusarium solani* mycelia and root rot (Cano-Salazar et al., 2011). During a seven-month field trial, the endophytic *B. cenocepacia* reduced the prevalence of fusarium wilt disease in banana plants by 3.4%, compared to 24.5% in untreated infected plants (Sapak et al., 2008). The antibiotic Pyrrolnitrin, which helps to reduce cotton damping off losses brought on by *Rhizoctonia solani*, was developed by several endophytic *Pseudomonas fluorescens* strains (Timper et al., 2009). *Fusarium oxysporum*, which was used as a bio-agent to create resistance in tomato plants, was successfully protected against *P. fluorescens* in flowering plants (Dudai, 2011). A bacteria that inhabit plant roots called *Bacillus amyloliquefaciens* has the power to control plant diseases and promote plant growth (Vardi et al., 2021).

In a study, it was discovered that bacterial endophytes shield cucumber plants from the cucumber anthracnose produced by *Pseudomonas fluorescents* (Akköprü et al., 2021). It was once believed that *Achromobacter* sp.*, Streptomyces* sp., and *Bacillus licheniformis* were responsible for the foliar disease known as downy mildew. The downy mildew disease infestation level was lowered by *Pseudoperonospora cubensis* (Basu et al., 2022), which ultimately resulted in an increased yield.

**Figure 3:**
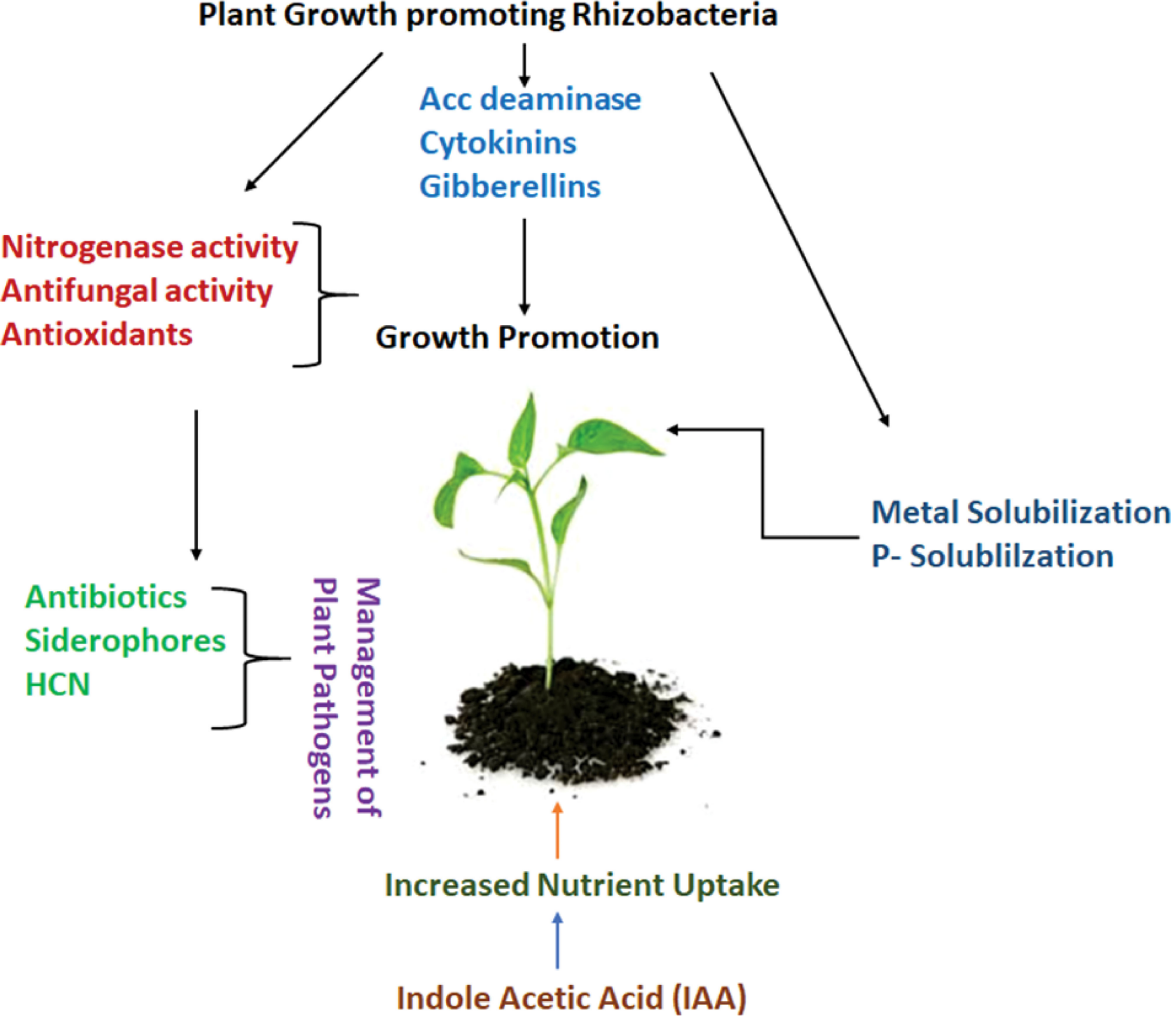
Mechanism of plant growth promotion by rhizobacteria (PGPR).

The management of pests, which has become a challenge for most crops since pests have evolved a tolerance to pesticides, is another use for these endophytic bacteria (Deng et al., 2014). Entomopathogenic bacteria have been used to combat pests that are immune to insecticides (**Figure 3**). A few fungi from the genera *Podonectria, Verticillium, Hirsutella, Sphaerostilbe, Agerata, Metarhizium Aschersonia*, and *Myriangium* are used for the biological management of pests. *Brevibacillus laterosporu*s is effective against nematodes, Lepidoptera, Coleoptera, and toxic fungi in plants in addition to insects (Skinner et al., 2014).

## 5 Identification of endophytic bacteria interaction with Host

In recent years, next-generation sequencing (NGS) techniques have been utilized to study the whole population of cultivable or non-cultivable bacteria inside plants, as well as their genomes. The interaction of host and bacterial endophytes has insightful concerns for the biological functioning of plants. As a result of interactions, rapid changes in host phenotype occurs also it is assumed as a driving force for the speciation and co-evolution of both the genetic system of host and bacteria (Fawcett, 1944). Though old genetic techniques to study plant-microbe interaction are less efficient, time-consuming, costly, and labor-intensive required a wide range of experiments and are usually limited to certain known genes (De Oliveira et al., 2004) in comparison to investigating the host-microbe interactions in molecular levels, it is needed to understand the phenotypic phenomena and genomics in depth. So the development of NGS technologies or metagenomic studies has provided the best way to understand the host-pathogen system. Through this technology, we can construct genome models of different organisms, which includes strains, their natural populations over time and their evolutionary histories (Navas et al., 2017; Sharma et al., 2021).

These complicated interactions can be analyzed and integrated by viewing plant microbiomes as a system. To better understand endophytism, contemporary genomic investigations incorporating metaomics and comparative studies can be quite beneficial (Dubey et al., 2020). A better understanding of endophyte interactions could be used to improve agricultural management by increasing plant development, biocontrol, and bioremediation (Alaimo et al., 2018). Some of the tools being utilized or that could be used to understand the link between plants and endophytes include genome sequencing, comparative genomics, microarray, next-generation sequencing, metagenomics, and metatranscriptomics (Dixit et al., 2022). To study endophytes and their apparent function in host plant ecology, contemporary methods and approaches need to be investigated (Gaiero et al., 2013).

Another way to identify the endophytic bacteria interact with the plant is to isolate the endophytic bacteria culture and then classify based on its phenotypic traits, and a few isolates from each category are identified further through partial sequencing of the 16S rRNA gene (Khare et al., 2018). The results of partial sequencing show that the isolates belonged to the genera *Pseudomonas*, *Stenotrophomonas, Bacillus, Pantoea*, and *Serratia* of bacteria (Liu et al, 2017>2552). These isolates are tested for their ability to produce siderophores, phosphate solubilization, atmospheric nitrogen fixation, protease, and hydrogen cyanide, as well as phytohormones like auxin and gibberellin (Eid et al., 2019). Auxin and gibberellin, two plant growth hormones, can be produced by all strains, though to varying degrees. Almost all strains could solubilize phosphate (Lata et al., 2019). The outcomes of protease, siderophore, and atmospheric nitrogen-fixing ability vary between strains. These findings provide information on the relationship between endophytic bacteria and their host plant (Vandana et al., 2021).

Furthermost genomic methods require recognition of variations among sequences within species or populations, like point mutations, Addition/deletions, and structural variations in structures (Bulgarelli et al., 2013).

### 5.1 Evolution of new pathogenic strains of microbes

One of the great evolutionary changes in life is the development of advantageous symbioses between eukaryotic (plants) and prokaryotic creatures (Chebotar et al., 2015). According to certain theories, the relationship between endophytic bacteria and plants frequently depends on two fundamental elements: currency and a system for exchanging currency. The currency could be, for instance, a root exudate that bacteria can take up in the context of interactions between plants and endophytic bacteria (Mercado-Blanco and JJ Lugtenberg, 2014). Similarly, bacteria may release hormones that encourage plant growth, such as auxin and gibberellins, which may be favorable for plant growth (Maksimov et al., 2018). It is anticipated that selection will favor the evolution of mutualism when the exchange of currencies between the two parties is balanced. Therefore, it is hypothesized that increased mutualistic dependency develops through reciprocal co-evolution or adaptation by one of the partners through the selection of features directly related to the mutualistic interaction (Chen et al., 2021).

Competition for scarce shared resources like iron may also lead to asymmetrical currency exchange, which could help to explain why some plant-microbe interactions are hostile (Hong and Park, 2016). Furthermore, because the rhizosphere is open, the free diffusion of resources derived from plants may promote higher levels of cheating in which mutant bacterial genotypes take benefit of “public goods” without producing substances that aid plant growth (Pandey et al., 2017). Because of this, mutualistic plant-microbe interactions may need additional enforcement from the plant, such as penalizing dishonest bacterial genotypes or positively identifying genotypes that promote plant growth (Ryan et al., 2008).Intriguing research would also be done to see whether endophytic bacteria and plants may coevolve from first neutral interaction and whether plants can coevolve in response to rhizosphere bacteria (Santos et al., 2018). In conclusion, by showing that plant-associated bacteria can quickly evolve along the symbiotic connection within a few growth cycles, our results urge eco-evolutionary management of endophytic bacteria and plants interactions in agriculture (Aswani et al., 2020).

### 5.2 Endophytic bacteria in disease management

Crop productivity is impacted by a number of common plant diseases that are present worldwide. Some of the serious ones are wilt disease, root rot, powdery mildew, leaf spot, leaf curl, and blight. To counter these phytopathogens, endophytic bacteria are crucial (Latha et al., 2019).

By producing proteins associated with pathogenesis (PRPs) and defense enzymes that stop the growth of phytopathogens that cause disease, endophytic bacteria can produce siderophores, antimicrobial compounds, and systemic resistance (Pandey et al., 2019). Bacterial endophytes are also potentially useful biocontrol agents. Plant diseases degrade plant performance and crop quality, which reduces crop output (Muthukumar et al., 2017). It has been shown that the nitrogen-fixing bacteria *Azotobacter chrococcum*, the phosphate-solubilizing bacteria PSB (*Pseudomonas cepacia*), the endophytic bacterial strains *Lysinibacillus* sp. and *Bacillus subtilis*, and their combination as bio-fertilizers can reduce the incidence of bacterial wilt disease in chili plants by up to 80% (Tewari et al., 2019).

The endophytic bacterial strain *B. subtilis* showed the strongest (80%) illness suppression (Jacob et al., 2020). This endophyte could also considerably aid the growth of the chili. Chemical pesticides are typically used to manage such phytopathogens, but this tactic has raised concerns about environmental contamination and contributed to the emergence of resistance to specific chemicals over time (Prasad et al., 2020). New insecticides must always be developed to address this. Chemical pesticides are thought to be ineffective when compared to endophytic bacteria acting as biocontrol agents or bioinsecticides. A broad array of mechanisms, including direct antagonism *via* the generation of antibiotics, siderophores, hydrogen cyanide, hydrolytic enzymes (chitinases, proteases, and lipases), etc., are involved in the biocontrol of plant diseases (Puri et al., 2017).

## 6 Conclusion

Some of the bacterial endophytes or PGPR are commonly used to control different diseases and as biological control agents so nowadays most of the focus is the understanding of complex interactions and their mechanisms and outcome either beneficial or harmful. It is hard to find the exact mechanism of interaction among complex microbial populations residing in the soil and environment near to host. So that proper characterization and management strategies can be devised according to the current need of time. In recent time peoples are preferring organic food and disliked the use of fertilizers and chemicals in agriculture. As the world population is increasing and food shortage issues are raised, in the current situation food security is an important topic for debate. Hence bacterial endophytes can be used as an alternative to chemical fertilizers, nutrient sources, and biological control agents for various plant pathogens. Scientists are focusing on the use of these endophytes in the form of biopesticides, and biofertilizers with different trade names for the control of different diseases and sustainable agricultural systems. Although the application of these endophytes in combination may lead to the development of optimum PGPEs inoculants that robust, and slight variation of environmental factors will not affect the plant growth promotion.

## Author contributions

SM, MN, MH, MS, and MA conceived and conceptualized the study. MAS, AS, MB provided materials and technical assistance. SM, MS wrote original draft. SS, MSH, MS and MT technically reviewed and finalized the draft. All authors contributed to the article and approved the submitted version.
